# Dataset: Ecosystem services and uses of dune systems of the coast of the Araucanía Region, Chile: A perception study

**DOI:** 10.1016/j.dib.2021.106725

**Published:** 2021-01-20

**Authors:** Pablo Arévalo-Valenzuela, Fernando Peña-Cortés, Jimmy Pincheira-Ulbrich

**Affiliations:** a Magíster en Planificación y Gestión Territorial, Universidad Católica de Temuco, Rudecindo Ortega 02950, Chile; b Laboratorio de Planificación Territorial, Departamento de Ciencias Ambientales, Facultad de Recursos Naturales, Universidad Católica de Temuco, Rudecindo Ortega 02950, Chile; c Laboratorio de Planificación Territorial, Departamento de Ciencias Ambientales, Facultad de Recursos Naturales, Universidad Católica de Temuco, Rudecindo Ortega 02950, Chile

**Keywords:** Dune fields, Interviews, Ecosystem services, Indigenous communities, Local actors

## Abstract

The dataset shows the relationship and valuation of the coastal dunes of the Araucanía region in Chile. The valuation of the local population was surveyed using a questionnaire applied to 49 subjects belonging to Mapuche communities and local government. The data consists of eight tables that show a list of questions, the number of times per year that visit the dunes, cultural practices carried out in the dunes, valuation of ecosystem services provided by the coastal dunes, and knowledge about flora and fauna. Lastly, the original questionnaire and its responses in Spanish and English are included in supplemantary material. This dataset was generated within the framework of the manuscript “Ecosystem services and uses of dune systems of the coast of the Araucanía Region, Chile: a perception study” where 23 leaders of Mapuche communities and 26 representatives of the local government were interviewed. The dataset can be used to compare the valuation of ecosystems by local communities, especially when quantitative data are scarce or do not exist.

## Specifications Table

**Table:** 

Subject	Social sciences
Specific subject area	Geography, Planning and Development. The data is part of the knowledge of local actors. This knowledge is the expression that emerges from the interaction between people and their environment, which is the basis for participatory planning.
Type of data	Table
How data were acquired	Semi-structured survey
Data format	Raw Analyzed data
Parameters for data collection	The main criterion for the data collection was the selection of participants who knew the dunes or responsibility in making decisions. In this framework, Mapuche communities – adjacent to the dune fields – and representatives of the local government were selected.
Description of data collection	The valuation of the local population was surveyed using a questionnaire applied to 49 subjects belonging to Mapuche communities and representatives of the local government. The questionnaire and its responses in Spanish and English are included in supplemantary material.
Data source location	Institution: Laboratorio de Planificación Territorial, Universidad Católica de Temuco. City/Town/Region: Temuco/La Araucanía Country: Chile Latitude and longitude (and GPS coordinates) for collected samples/data: The area covered by dunes is 4,349 ha, distributed in eight dune fields between the Imperial river to the north (38° 44’S, 73° 27’W) and the Queule river in the south (39° 24’S, 73° 09’W).
Data accessibility	With the article
Related research article	P. Arévalo-Valenzuela, F. Peña-Cortés, J. Pincheira-Ulbrich, Ecosystem services and uses of dune systems of the coast of the Araucanía Region, Chile: A perception study, Ocean & Coastal Management. 200 (2020) 1–10 [1]. https://doi.org/10.1016/j.ocecoaman.2020.105450.

## Value of the Data

• The data provide the identification and valuation of the ecosystem services of the coastal dunes, a topic with a scarce record on a global scale.
• The data shows cultural practices of the Mapuche people in a poorly studied environment.
• The data could be used to evaluate or implement public policies in a local context. It also allows comparing local governance in similar contexts.
• Data on perception and local knowledge are especially important when there is no other source of data.

## 1 Data Description

The data consists of eight tables in which the perception and local knowledge are collected from the Mapuche communities and representatives of the local government. Table 1 present the questionnaire by question type of the related manuscript. Table 2 shows the number of times per year in which the dunes are visited. Table 3 provides a summary of the quantitative results by question type. Table 4 show cultural practices carried out in the dunes. Table 5, 6 and 7 present the valuation of the ecosystem services provided by the coastal dunes; and Table 8, shown the summary of answers to open questions about the species of flora and fauna. Lastly, the original questionnaire and its responses in Spanish and English are included in supplemantary material.

**Table 1:** Matrix of the sections in the questionnaire, with the questions divided by dimensions. X denotes the selected category, – Not applicable.

		Type of question			
Dimension	Statement	Binary	Multiple choice	Open	Likert
Use and management	1. Have you visited the dune fields? How many times per year do you go?	X	–	X	–
	2. What benefits do the dunes provide for the coast?	–	–	X	–
	3. What is the degree of use of the dunes? a) Very low, b) Low, c) Medium, d) High, e) Very high.	–	–	–	X
	4. What activities are carried on by the residents of local communities in the dunes and neighbouring areas? a) Tourism, b) Agriculture, c) Forestry, d) Stock-raising, e) Conservation	–	X	–	–
	5. What is the principal type of use given to dunes and their environment by non-local people? a) Tourism, b) Agriculture, c) Forestry, d) Stock-raising, e) Conservation	–	X	–	–
	6. What is the best use that could be given to the dunes? a) Conservation, b) Recreation and tourism, c) Economic-productive activities (e.g. farming, stock-raising, etc.), d) A combination of conservation and tourism.	–	X	–	–
	7. Do you think that use of the dunes is causing their deterioration?	X	–	–	–
Valuation	8. Do you know about the concept of ES? If so, explain it in your own words.	X	–	X	–
	9. Evaluate the potential importance of the dunes as providers of benefits. a) Very low, b) Low, c) Medium, d) High, e) Very high.	–	–	–	X
	10. Assess the following sub-categories of ES provided by the dunes on a scale of 1 to 5: a) Habitat for flora and fauna, b) Production or Socio-economic, c) Sand reserve, d) Space for ore deposit, e) Space for accumulating material, f) Regulation of natural process, g) Absorbing wave energy, h) Water resource regulation, i) Information and culture, j) Identity (sense of place), k) Space for human activities, l). Space with aesthetic value.	–	–	–	X
	11. Do you think the restoration and/or maintenance of the dunes is important?	X	–	X	–
Cultural – Political	12. Which are the main human groups who benefit from the dunes? a) Local residents, b) Small-scale farmers, c) Artisanal fishermen, d) Mapuche communities, e) Tourists and tourist businesses	–	X	–	–
	13. What are the main socio-territorial problems involving the dunes? a) Conflicts over land ownership, b) Extraction of materials and sand/gravel, c) Tourism v/s agriculture and/or stock-raising, d) Natural hazard (storms, storm surge, heavy swell, tsunami), e) None.	–	X	–	–
	14. Are there any cultural practices which take place in the dunes and surrounding areas? What are they?	X	–	X	–
	15. Have you heard of CONAF's initiative to create “natural vegetation barriers”, carried out in the dunes around Saavedra after the 2010 tsunami?	X	–	–	–
	16. Do you think that CONAF's initiative (Question 15) would help to mitigate possible natural events associated with storms, storm surge or heavy swell?	X	–	–	–
Biophysics	17. Do you know if any animals live in the dunes? Can you name them?	X	–	X	–
	18. Do you know if any plants grow in the dunes? Can you name them?	X	–	X	–
	19. If a natural event associated with storms, storm surge or heavy swell affected the coast of the Araucanía Region, would the dunes help at all to mitigate the possible impacts?	X	–	–	–
	20. Do you think that the dunes would help to mitigate possible natural events associated with a tsunami?	X	–	–	–

**Table 2:** Number of times per year that Mapuche communities and representatives of the local government visit the dunes (see statement 1 in related research article).

Respondent	Mapuche communities (times per year)	Representatives of the local government (times per year)
1	150	3
2	5	5
3	20	0
4	80	2
5	4	50
6	0	12
7	2	3
8	25	30
9	365	20
10	365	4
11	7	35
12	4	115
13	150	18
14	28	11
15	250	20
16	4	8
17	365	6
18	50	20
19	35	3
20	7	12
21	20	10
22	20	10
23	Respondent does not know	1
24	–	2
25	–	2
26		3
Average ($\tilde{x}$/year)	89	16
Standard deviation	128.39	23.55
Median	22.5	9

**Table 3:** Replies given to binary, multiple-choice and Likert scale questions by Mapuche communities and local government respondents. “Statement” as per Table 1. Map = Mapuche communities, Gov. = representatives of the local government. The figures shown for questions 4, 5, 6, 12 and 13 represent the frequency of response for each category; more than one category could be selected. –Not applicable.

		Binary						Multiple choice															Likert														
		Map.		Gov.		Total		Map.					Gov.					Total					Map.					Gov.					Total				
Dimension	Statement	yes	no	yes	no	yes	no	a	b	c	d	e	a	b	c	d	e	a	b	c	d	e	1	2	3	4	5	1	2	3	4	5	1	2	3	4	5
Use and management	1	22	1	25	1	47	2	–	–	–	–	–	–	–	–	–	–	–	–	–	–	–	–	–	–	–	–	–	–	–	–	–	–	–	–	–	–
	3	–	–	–	–	–	–	–	–	–	–	–	–	–	–	–	–	–	–	–	–	–	4	6	9	2	2	1	11	9	4	1	5	17	18	6	3
	4	–	–	–	–	–	–	15	18	11	19	1	24	0	7	10	5	39	18	18	29	6	–	–	–	–	–	–	–	–	–	–	–	–	–	–	–
	5	–	–	–	–	–	–	6	7	8	11	2	20	0	5	7	19	26	7	13	18	21		–	–	–	–	–	–	–	–	–	–	–	–	–	–
	6	–	–	–	–	–	–	4	2	0	17	–	8	3	0	15	–	12	5	0	32	–	–	–	–	–	–	–	–	–	–	–	–	–	–	–	–
	7	21	2	23	3	44	5	–	–	–	–	–	–	–	–	–	–	–	–	–	–		–	–	–	–	–	–	–	–	–	–	–	–	–	–	–
Valuation	8	1	22	10	16	11	38	–	–	–	–	–	–	–	–	–	–	–	–	–	–	–	–	–	–	–	–	–	–	–	–	–	–	–	–	–	–
	9	–	–	–	–	–	–	–	–	–	–	–	–	–	–	–	–	–	–	–	–	–	0	2	4	9	8	0	2	7	13	4	0	4	11	22	12
	11	23	0	24	2	47	2	–	–	–	–	–	–	–	–	–	–	–	–	–	–	–	–	–	–	–	–	–	–	–	–	–	–	–	–	–	–
Cultural – Political	12	–	–	–	–	–	–	17	11	18	18	10	17	5	20	18	25	34	16	38	36	35	–	–	–	–	–	–	–	–	–	–	–	–	–	–	–
	13	–	–	–	–	–	–	7	7	2	21	2	13	10	5	20	0	20	17	7	41	2	–	–	–	–	–	–	–	–	–	–	–	–	–	–	–
	14	21	2	15	11	36	13	–	–	–	–	–	–	–	–	–	–	–	–	–	–	–	–	–	–	–	–	–	–	–	–	–	–	–	–	–	–
	15	16	7	18	8	34	15	–	–	–	–	–	–	–	–	–	–	–	–	–	–	–	–	–	–	–	–	–	–	–	–	–	–	–	–	–	–
	16	13	3	15	3	6	28	–	–	–	–	–	–	–	–	–	–	–	–	–	–	–	–	–	–	–	–	–	–	–	–	–	–	–	–	–	–
Biophysics	17	23	0	26	0	49	0	–	–	–	–	–	–	–	–	–	–	–	–	–	–	–	–	–	–	–	–	–	–	–	–	–	–	–	–	–	–
	18	23	0	26	0	49	0	–	–	–	–	–	–	–	–	–	–	–	–	–	–	–	–	–	–	–	–	–	–	–	–	–	–	–	–	–	–
	19	23	0	26	0	49	0	–	–	–	–	–	–	–	–	–	–	–	–	–	–	–	–	–	–	–	–	–	–	–	–	–	–	–	–	–	–
	20	15	8	24	2	39	10	–	–	–	–	–	–	–	–	–	–	–	–	–	–	–	–	–	–	–	–	–	–	–	–	–	–	–	–	–	–

**Table 4:** Cultural practices carried out in the dunes recognized by Mapuche communities and representatives of the local government (see statement 14). *Llellipun*: ceremony or collective rogation that seeks to prepare integrally for a given event, *Machitun*: ceremony to cure diseases, *Nguillatun*: traditional ritual to pray to the spirits for the well-being, prosperity and balance of the community, Palin: traditional sport similar to hockey, *We tripantu*: celebration of the Mapuche New Year.

Respondent	Mapuche communities	Representatives of the local government
1	*Nguillatun*	Yes
2	*Nguillatun, Llellipun, We tripantu*	Respondent does not know
3	*Nguillatun*, horse races (yenehue sector)	Respondent does not know
4	*Nguillatun*	*Nguillatun, Machitun,* spaces for sports,
5	*Nguillatun*	Respondent does not know
6	*Nguillatun, Palin*, Machitun	Yes
7	*Nguillatun*	Respondent does not know
8	*Nguillatun*, collection of medicinal plants	Ceremonial sites for the Mapuche culture
9	Horse races	*Nguillatun*
10	Yes	Respondent does not know
11	*Nguillatun, Palin*.	*Nguillatun, Machitun*, spiritual rituals
12	*Palin, Chueca*, Horse races	Ceremonial activities
13	Respondent does not know	*Nguillatun*
14	*Nguillatun*	*Nguillatun, Wetripantu, Machitun, Palin, Llellipun*
15	Rogative*, Nguillatun, Palin*, horse races, bathe animals	Respondent does not know
16	Horse races, *We tripantu*	Yes
17	*Nguillatun,* rogative	Respondent does not know
18	Yes	Respondent does not know
19	*Nguillatun*	*Nguillatun*, Rogative
20	Rogative	Ceremonial sites
21	Respondent does not know	Respondent does not know
22	*Nguillatun*	Respondent does not know
23	Respondent does not know.	Respondent does not know
24	–	*Nguillatun*
25	–	*Nguillatun*
26	–	*–*

**Table 5:** Mapuche communities’ (n = 23) valuation of ecosystem services provided by the coastal dunes of the Araucanía Region. The categories were classified according to CISESS (Haines-Young et al. 2018) and subcategories according to Castro (2015) using a Likert scale: 1 = very low; 2 = low; 3 = medium; 4 = high; and 5 = very high (see statement 10).

	Provisioning					Regulation and maintenance			Cultural			
Respondent	Habitat for flora and fauna	Production or Socio-economic	Sand reserve	Space for storing ores	Space for accumulating material	Regulation of natural process	Absorbing wave energy	Water resource regulation	Information and culture	Identity (sense of place)	Space for human activities	Space with aesthetic value
1	5	4	5	1	5	5	5	5	5	5	4	5
2	3	4	5	1	1	5	5	2	4	3	2	5
3	5	5	3	1	5	5	5	4	5	4	5	5
4	5	5	4	1	5	4	5	3	5	5	5	4
5	4	3	3	1	5	5	5	1	5	5	5	5
6	2	1	1	1	3	4	3	1	4	5	4	5
7	4	3	4	1	3	3	3	1	3	4	4	3
8	5	5	3	1	5	5	5	1	5	5	5	5
9	2	3	2	1	1	5	5	5	5	5	1	5
10	5	1	5	1	1	5	5	4	2	5	1	5
11	4	1	2	1	1	4	5	4	2	4	4	4
12	5	2	5	5	1	5	5	5	5	5	5	5
13	4	4	5	1	5	5	5	5	3	4	5	5
14	4	3	3	1	1	5	5	1	5	5	5	5
15	4	3	4	1	4	5	5	1	3	5	4	4
16	4	1	1	1	1	2	1	2	4	4	3	5
17	5	1	5	1	1	5	5	4	5	5	5	5
18	5	4	3	1	4	4	5	1	4	2	5	5
19	4	4	5	1	1	5	5	3	4	5	4	5
20	2	5	5	1	1	4	5	1	5	5	5	5
21	5	5	4	1	5	5	5	1	5	4	5	5
22	4	4	5	1	4	5	5	1	5	5	3	5
23	5	5	3	1	5	5	4	4	5	5	5	5
Average	4.1	3.3	3.7	1.2	3.0	4.6	4.6	2.6	4.3	4.5	4.1	4.8
Mode	5.0	4.0	5.0	1.0	1.0	5.0	5.0	1.0	5.0	5.0	5.0	5.0
Standard deviation	1.0	1.5	1.3	0.8	1.8	0.8	1.0	1.6	1.0	0.8	1.3	0.5
Median	4.0	4.0	4.0	1.0	3.0	5.0	5.0	2.0	5.0	5.0	5.0	5.0

**Table 6:** Representatives of the local government (n = 26) valuation of ecosystem services provided by the coastal dunes of the Araucanía Region. Categories were classified according to CISESS (Haines-Young et al. 2018) and subcategories according to Castro (2015). Likert scale: 1 = Very Low; 2 = Low; 3 = Medium; 4 = High; and 5 = Very high (see statement 10).

	Provisioning					Regulation and maintenance			Cultural			
Respondent	Habitat for flora and fauna	Production or Socio-economic	Sand reserve	Space for storing ores	Space for accumulating material	Regulation of natural process	Absorbing wave energy	Water resource regulation	Information and culture	Identity (sense of place)	Space for human activities	Space with aesthetic value
1	4	1	4	1	1	3	5	1	1	3	4	4
2	4	5	3	1	3	4	5	1	2	3	4	3
3	2	3	3	3	2	5	4	5	2	4	2	1
4	4	3	4	4	4	5	5	1	5	5	4	4
5	5	3	3	1	4	5	5	2	3	2	4	3
6	5	2	4	1	3	5	4	1	3	2	3	3
7	5	3	5	4	4	2	5	5	3	5	4	5
8	4	4	4	1	4	5	4	1	4	3	3	4
9	1	2	5	3	1	5	1	1	4	5	5	1
10	4	4	1	1	1	3	5	1	4	4	5	4
11	4	2	5	1	2	3	5	1	2	5	5	5
12	2	3	5	1	1	5	5	1	2	5	4	3
13	4	4	5	1	2	5	5	1	2	5	5	5
14	5	2	5	4	4	5	3	2	3	2	4	4
15	5	2	5	1	5	4	5	5	3	5	1	5
16	5	5	4	3	4	5	5	5	4	5	5	5
17	5	5	5	1	3	4	5	1	4	5	5	5
18	2	4	1	1	1	5	3	1	5	2	2	4
19	4	3	3	1	1	5	4	1	5	1	3	3
20	5	2	5	1	2	5	5	1	1	4	3	5
21	4	4	5	3	4	5	5	1	3	4	3	5
22	5	3	4	4	4	5	5	1	1	5	5	5
23	2	2	5	1	2	5	5	1	3	3	2	4
24	5	4	5	5	5	5	5	5	4	4	4	4
25	3	3	5	3	2	3	5	5	1	1	4	2
26	3	3	5	3	2	4	4	5	5	1	4	2
Average	3.88	3.12	4.15	2.08	2.73	4.42	4.50	2.15	3.04	3.58	3.73	3.77
Mode	5	3	5	1	4	5	5	1	3	5	4	5
Standard deviation	1.21	1.07	1.19	1.35	1.34	0.90	0.95	1.78	1.31	1.45	1.12	1.24
Median	4	3	5	1	3	5	5	1	3	4	4	4

**Table 7:** Valuation categories of the ecosystem services provided by the coastal dunes. Statement: Assess the following sub-categories of ES provided by the dunes on a scale of 1 to 5. Categories were classified according to CISESS (Haines-Young et al. 2018) and subcategories according to Castro (2015). Likert scale: 1= very low; 2= low; 3= medium; 4= high; and 5= very high. G-test = Goodness-of-fit test under the null hypothesis of no difference between the responses categories and the average (Mean, [H0]; p≥0.05). Bailey's simultaneous confidence intervals: frequency lower (*) or higher (**) than expected by chance (Mean, [H0]; p≥0.05). The sample size was considered sufficient when at least the second Cherry test was passed. Categories: 1.1. Habitat for flora and fauna, 1.2. Production or Socio-economic, 1.3. Sand reserve, 1.4. Space for ore deposit, 1.5. Space for accumulating material, 2.1. Regulation of natural process, 2.2. Absorbing wave energy, 2.3. Water resource regulation, 3.1. Information and culture, 3.2. Identity (sense of place), 3.3. Space for human activities, 3.4. Space with aesthetic value.

	Mapuche communities (n=23)					G-test		Cherry's doubletest		Local government (n=26)					G-test		Cherry's doubletest	

Ecosystem Services Categories and subcategories	Verylow	Low	Medium	High	Veryhigh	Mean(H0)	p	1^st^Npj	2^nd^N(1−pj)	Verylow	Low	Medium	High	Veryhigh	Mean(H0)	p	1^st^Npj	2^nd^N(1−pj)
1. Provisioning																		
1.1. Habitat for flora and fauna	0*	3	1*	9	10	4.60	<0.001	No	Yes	1	4	3	9	9	5.2	0.026	No	yes
1.2. Production or Socio-economic	5	1	5	6	6	4.60	0.288	No	Yes	1	6	9	7	3	5.2	0.057	No	yes
1.3. Sand reserve	2	2	6	4	9	4.60	0.112	No	Yes	2	0*	4	7	13**	5.2	<0.001	No	yes
1.4. Space for storing ores	22**	0*	0*	0*	1	4.60	<0.001	No	No	16**	1	5	3	1	5.2	<0.001	No	yes
1.5. Space for accumulating material	10	0	2	3	8	4.60	0.001	No	Yes	6	6	4	8	2	5.2	0.354	No	yes
2. Regulation and maintenance																		
2.1. Regulation of natural process	0*	1	1	5	16**	4.60	<0.001	No	Yes	0*	1	4	5	16**	5.2	<0.001	No	yes
2.2. Absorbing wave energy	1	0*	2	1	19**	4.60	<0.001	No	No	1	0*	2	4	19**	5.2	<0.001	No	yes
2.3. Water resource regulation	10	2	2	5	4	4.60	0.052	No	Yes	18	2	0	0	6	5.2	<0.001	No	yes
3. Cultural 3.1. Information and culture	0*	2	3	5	13**	4.60	<0.001	No	Yes	4	5	7	6	4	5.2	0.863	No	yes
3.2. Identity (sense of place)	0*	1	1	6	15**	4.60	<0.001	No	Yes	2	3	5	6	10	5.2	0.122	No	yes
3.3. Space for human activities	2	1	2	6	12**	4.60	0.001	No	Yes	1	3	5	9	8	5.2	0.044	No	yes
3.4. Space with aesthetic value	0*	0*	1	3	19**	4.60	<0.001	No	No	2	1	5	7	11	5.2	0.011	No	yes

**Table 8:** Summary of answers to open questions about the species of flora and fauna known to the Mapuche communities and local government representatives. * native species, ** introduced species. n= number of respondents.

Dimension	Statement	Identified species	Mapuche communities (n)	Local government (n)
Biophysics	17. Do you know if any animals live in the dunes? Can you name them?	Duck (*Anas ssp.*)*	1	0
		Turkey Vulture (*Cathartes aura*)*	1	1
		Pilpilen (*Haematopus palliatus*)*	1	0
		Seagull (*Larus spp.*)*	9	9
		Hare (*Lepus capensis*)**	8	8
		Lizard (*Liolaemus spp*.)*	0	2
		Fox (*Lycalopex spp.*)*	5	2
		Coipo (Myocastor coypus)*	1	0
		Rabbit (*Oryctolagus cuniculus*)**	8	7
		Cormorant (*Phalacrocorax gaimardi*)*	3	0
		Snake (*Philodryas chamissonis* or *Tachymenis chilensis*)*	1	0
		Treile (*Vanellus chilensis*)*	4	0
		No reply	4	9
	18. Do you know if any plants grow in the dunes? Can you name them?	Acacio (*Acacia melanoxylon*)**	1	0
		Marram grass (*Ammophila arenaria*) **	5	9
		Ratonera (*Anthoxanthum utriculatum*)*	1	1
		Apio de mar (*Apium australe*)*	1	0
		Michay (*Berberis spp.*)*	2	0
		Doca (*Carpobrotus chilensis*)*	1	3
		Monterey cypress (*Cupressus macrocarpa*)**	1	0
		Blue gum (*Eucalyptus spp.*)**	1	1
		Pichoga (*Euphorbia helioscopia*)**	1	0
		Native strawberry (*Fragaria chiloensis*)*	0	2
		Chupon (*Greigia sphacelata*)*	1	0
		Nalca (*Gunnera tinctoria*)*	1	0
		Perlilla (*Hedyotis salzmannii*)*	2	0
		Rushes (*Juncus procerus*)*	1	1
		Chocho (*Lupinus arboreus*)**	5	6
		Mailhuén (*Maihuenia poeppigii*)*	8	1
		Boldo (*Peumus boldus*)*	1	0
		Pine (*Pinus radiata*)**	2	8
		Sanguinarea (*Polygonum maritimum*)*	9	1
		Totora (Typha spp.)*	0	1
		Murtilla (*Ugni spp*.)*	3	1
		Picapica (*Ulex europaeus*)**	1	0
		No reply	4	8

## 2 Experimental Design, Materials and Methods

The database was built in the dune systems located in the *comunas* (municipal districts) of Saavedra, Teodoro Schmidt and Toltén on the coast of the Araucanía Region, Chile. The area covered by dunes is 4,349 ha (Peña-Cortés et al. distributed in eight dune fields between the Imperial river to the north (38° 44’S, 73° 27’W) and the Queule river in the south (39° 24’S, 73° 09’W).

The coast of the Araucanía Region presents the highest percentages of poverty and rural habitation in Chile (CASEN 2015), resulting from high levels of land division (small plot size), a farming-based economy, and in recent years the impact of forestry plantations Peña-Cortés et al., as a result of which there are few remnant patches of native forest Pincheira-Ulbrich et al.

The subsistence farming economy is associated with Mapuche communities and rural inhabitants Peña-Cortés et al. As with the rest of the country, most major decisions are taken centrally in the national capital, Santiago, although a certain level of decision-making occurs at local government level, in the towns of Saavedra, Toltén and Teodoro Schmidt where most public services are concentrated.

The study participants were selected using the official lists of Mapuche communities and local government employees. In the case of the Mapuche group (community presidents), the information was provided by the National Indigenous Development Corporation (CONADI), the Local Development Programme (PRODESAL) and the Intercultural Departments of the respective municipality. The information on local government personnel was obtained from the websites of the municipalities of Teodoro Schmidt (http://muniteodoro.cl/portal/), Saavedra (http://municipiodesaavedra.cl/Municipio/), and Toltén (http://www.tolten.cl/web/). The selection strategy for the local government group targeted most of the heads of services directly related with the natural system, namely: the Local Development Unit (UDEL), the Community Development Office (DIDECO), the Planning Secretary (SECPLAC), the Territorial Development Programme (PDTI) and the Environment Department. Initial contact with this group was by e-mail, allowing interviews to be coordinated. Local government actors were selected in this way. The participants of the Mapuche group were selected following a non-probabilistic strategy through a “snow-balling” procedure; the first contact was made with the President of the Association of Lafquen Leufu Communities (Saavedra), and a total sample of 23 people was obtained (Vieytes, 2004).

The perceptions of the actors were obtained by a questionnaire applied individually between January and February 2018, in a dialogue lasting approximately 30 to 60 min. There were 11 binary questions (yes-no), five multiple choice (five or six options per question), seven open questions and three using a Likert-type scale. The Likert scale questions used a scale of one to five (e.g. 1 = Very Low; 2 = Low; 3 = Medium; 4 = High; and 5 = Very high), covering the full range of values for the item being assessed. Questions of this type included the classification categories proposed by Castro (2015) which are then presented in results rearranged according to Common International Classification of Ecosystem Services (CISES) (Haines-Young et al. 2018) (Questions 10). Each section included an explanation of the main concepts of the theme, to ensure correct assessment by the participants.

For multiple-choice question in Table 7 G-test goodness of fit was carried out. This allowed the relative proportions of the observed and expected frequencies to be compared, response by response, between the two groups (Mapuche communities and representatives of the local government) under the null hypothesis of no significant differences between the observed and the expected value (Krebs, 1989; Montenegro and Acosta, 2008). Considering that some response categories resulted in frequencies lower than five and even equal to zero, Cherry's double test was applied to determine the intervals for enough sample to perform a reliable analysis (Cherry, 1996).

## Ethics Statement

During the interview, efforts were made to maintain openness and non-judgmentality, treating each interviewee fairly and impartially, regardless of their socio-economic status, social status, race, religion or physical appearance. Potential research participants were asked to read the plain language statements and complete a research consent form if they are willing to participate in this project voluntarily. Potential participants were asked to read the research consent form in order to voluntarily express their willingness to participate in this project.

## CRediT Author Statement

**Pablo Arévalo-Valenzuela**: Conceptualization, Investigation, Application of method, Data analysis; **Fernando Peña-Cortés**: Supervision, Data discussion; **Jimmy Pincheira-Ulbrich**: Writing-Original draft preparation, Visualization, Reviewing and Editing, Data analysis.

## Declaration of Competing Interest

The authors declare that they have no known competing financial interests or personal relationships which have, or could be perceived to have, influenced the work reported in this article.

## Appendix Supplementary materials

Image, application 1
