# Supplementary material for: Dataset: Ecosystem services and uses of dune systems of the coast of the Araucanía Region, Chile: A perception study
Source: Data Brief. 2021 Jan 20;35:106725. doi: 10.1016/j.dib.2021.106725 (PMC7851771; doi:10.1016/j.dib.2021.106725)
Supplement: Supplementary file 1 [file mmc1.zip › Supplementary_file_questionnaire.docx]

Survey: Spanish translation of the questionnaire

**QUESTIONNAIRE**

**Aim of the questionnaire**: To find out the perception of the public-municipal sector and the Mapuche communities regarding the benefits provided by the dunes of the Araucanía coast.

The questionnaire is divided into five dimensions to facilitate its reading, covering from the general to the particular. The first section corresponds to the background of the respondent, the second section refers to the use and management of the dunes, the third section deals with the assessment of the dunes, the fourth section evaluates the cultural-political value of the dunes, and finally the fifth section deals with the biophysical dimension.

1. **BACKGROUND OF THE RESPONDENT**

| Name of the institution or organization to which it belongs. | |  | | |
| --- | --- | --- | --- | --- |
| Labour dependency sector | |  | Regional public service | |
|  |  |  | Municipality | |
|  |  |  | Company | |
|  |  |  | Non-governmental organization | |
|  |  |  | University/Research Centre | |
|  |  |  | Self-employed | |
| Commune |  | | | |
| Position (job title) |  | | | |
| Gender (M-F) |  | | | |
| Ethnic origin (Mapuche-chilean) |  | | | |
| Years of knowledge of the area |  | Years of experience in the current position | |  |

1. **USE AND MANAGEMENT OF DUNES**

| 1. Have you visited the dune fields? How many times per year do you go? | |
| --- | --- |
| a) Yes ___  b) No ___  Anwer:______________________________________________________________________  ______________________________________________________________________ | |
| 2. What benefits do the dunes provide for the coast? |  |
| Answer: ______________________________________________________________________  _____________________________________________________________________ |  |

| 3. What is the degree of use of the dunes? | | | | | |
| --- | --- | --- | --- | --- | --- |
| Likert scale | Very low | Low | Medium | High | Very high |
| Use |  |  |  |  |  |

| 4. What activities are carried on by the residents of local communities in the dunes and neighbouring areas? |
| --- |
| a) Tourism ___  b) Agriculture ___  c) Forestry ___  d) Stock-raising ___  e) Conservation ___ |

| 5. What is the principal type of use given to dunes and their environment by non-local people? |
| --- |
| a) Tourism ___  b) Agriculture ___  c) Forestry ___  d) Stock-raising ___  e) Conservation ___ |

| 6. What is the best use that could be given to the dunes? |
| --- |
| a) Conservation ___  b) Recreation and tourism ___  c) Economic-productive activities (e.g. farming, stock-raising, etc.) ___  d) A combination of conservation and tourism ___ |

| 7. Do you think that use of the dunes is causing their deterioration? |
| --- |
| a) Yes ___  b) No ___ |

1. **VALUATION OF ECOSYSTEM SERVICES**

| 8. Do you know about the concept of ES? If so, explain it in your own words. |
| --- |
| a) Yes ___  b) No ___  Answer:______________________________________________________________________  ______________________________________________________________________ |

| 9. Evaluate the potential importance of the dunes as providers of benefits | | | | | |
| --- | --- | --- | --- | --- | --- |
| Likert scale | Very low | Low | Medium | High | Very high |
| Importance |  |  |  |  |  |

| 10. Assess the following sub-categories of ES provided by the dunes on a scale of 1 to 5 | | | | | | |
| --- | --- | --- | --- | --- | --- | --- |
| Category | Subcategories | Likert scale | | | | |
|  |  | 1 | 2 | 3 | 4 | 5 |
| Provisioning | Habitat for flora and fauna |  |  |  |  |  |
|  | Production or Socio-economic |  |  |  |  |  |
|  | Sand reserve |  |  |  |  |  |
|  | Space for storing ores |  |  |  |  |  |
|  | Space for accumulating material |  |  |  |  |  |
| Regulation and maintenance | Regulation of natural process |  |  |  |  |  |
|  | Absorbing wave energy |  |  |  |  |  |
|  | Water resource regulation |  |  |  |  |  |
| Cultural | Information and culture |  |  |  |  |  |
|  | Identity (sense of place) |  |  |  |  |  |
|  | Space for human activities |  |  |  |  |  |
|  | Space with aesthetic value |  |  |  |  |  |

| 11. Do you think the restoration and/or maintenance of the dunes is important? |
| --- |
| a) Yes ___  b) No ___  Answer:______________________________________________________________________  ______________________________________________________________________ |

1. **CULTURAL-POLITICAL VALUATION**

| 12. Which are the main human groups who benefit from the dunes? |
| --- |
| a) Local residents ___  b) Small-scale farmers ___  c) Artisanal fishermen ___  d) Mapuche communities ___  e) Tourists and tourist businesses ___ |

| 13. What are the main socio-territorial problems involving the dunes? |
| --- |
| a) Conflicts over land ownership ___  b) Extraction of materials and sand/gravel ___  c) Tourism v/s agriculture and/or stock-raising ___  d) Natural hazard (storms, storm surge, heavy swell, tsunami) ___  e) None ___ |

| 14. Are there any cultural practices which take place in the dunes and surrounding areas? |
| --- |
| a) Yes ___  b) No ___  Answer: _____________________________________________________________________  _____________________________________________________________________ |

| 15. Have you heard of CONAF's initiative to create “natural vegetation barriers”, carried out in the dunes around Saavedra after the 2010 tsunami? |
| --- |
| a)Yes___  b) No ___ |

| 16. Do you think that CONAF's initiative would help to mitigate possible natural events associated with storms, storm surge or heavy swell? |
| --- |
| a) Yes ___  b) No ___ |

1. **BIOPHYSICAL AND ECOLOGICAL VALUATION**

| 17. Do you know if any animals live in the dunes? Can you name them? |
| --- |
| Answer: ______________________________________________________________________  ______________________________________________________________________ |
| 18. Do you know if any plants grow in the dunes? Can you name them? |
| Answer: ______________________________________________________________________  ______________________________________________________________________ |

| 19. If a natural event associated with storms, storm surge or heavy swell affected the coast of the Araucanía Region, would the dunes help at all to mitigate the possible impacts? |
| --- |
| a) Yes ___  b) No ___ |

| 20. Do you think that the dunes would help to mitigate possible natural events associated with a tsunami? |
| --- |
| a) Yes ___  b) No ___ |

Survey in original language (Spanish)

**CUESTIONARIO**

**Objetivo del cuestionario:** Conocer la percepción del sector Público–Municipal y las Comunidades Mapuches, respecto de los beneficios que proveen las dunas de la costa de La Araucanía.

El cuestionario se divide en cinco dimensiones para facilitar su lectura, desde lo general a lo particular. La primera sección corresponde a los antecedentes del entrevistado, la segunda sección hace referencia al uso y manejo de las dunas, la tercera sección trata sobre la valoración de las dunas, la cuarta sección evalúa el valor cultural-político de las dunas y finalmente la quinta, la dimensión biofísica.

1. **ANTECEDENTES DEL ENTREVISTADO**

| Nombre de la institución u organización a la que pertenece | |  | | |
| --- | --- | --- | --- | --- |
| Sector de dependencia laboral | |  | Servicio público regional | |
|  |  |  | Municipio | |
|  |  |  | Empresa | |
|  |  |  | ONG | |
|  |  |  | Universidad/Centro Investigación | |
|  |  |  | Trabajador por cuenta propia | |
| Comuna |  | | | |
| Cargo que desempeña |  | | | |
| Género (M-F) |  | | | |
| Origen etnico (Mapuche-chileno) |  | | | |
| Años de experiencia en el área |  | Años de experiencia en el cargo actual | |  |

1. **USO Y MANEJO DE LAS DUNAS**

| 1. ¿Ha visitado los campos de dunas? ¿Cuántas veces al año? |
| --- |
| a) Si ___  b) No ___  R:______________________________________________________________________  ______________________________________________________________________ |

| 2. ¿Cuáles son los beneficios que proporcionan las dunas para el borde costero? |
| --- |
| R: ______________________________________________________________________  _____________________________________________________________________ |

| 3. Según su apreciación ¿Cuál es el grado de uso de las dunas? | | | | | |
| --- | --- | --- | --- | --- | --- |
|  | Nula | Baja | Media | Alta | Máxima |
| Dunas |  |  |  |  |  |

| 4. ¿Qué actividades llevan a cabo los residentes de las comunidades locales en las dunas y areas aledañas? |
| --- |
| a) Turismo ___  b) Agricultura ___  c) Forestal ___  d) Ganadería ___  e) Conservación ___ |

| 5. ¿Cuál es el tipo principal de uso que las personas no-locales dan a las dunas y su entorno? |
| --- |
| a) Turismo ___  b) Agricultura ___  c) Forestal ___  d) Ganadería ___  e) Conservación ___ |

| 6. ¿Cuál es el mejor uso que se le podría dar a las dunas? |
| --- |
| a) Conservación ___  b) recreación y turismo ___  c) Actividades económico-productivo (agrícola, ganadero, entre otros) ___  d) Una combinación de conservación y turismo ___ |

| 7. ¿Cree que el uso de las dunas está causando su deterioro? |
| --- |
| a) Si ___  b) No ___ |

1. **VALORACION DE LAS DUNAS**

| 8. ¿Conoce el concepto de Servicios Ecosistémicos? Si es así, ¿Puede describirlo con sus propias palabras? |
| --- |
| a) Si ___  b) No ___  R:______________________________________________________________________  ______________________________________________________________________ |

| 9. Evaluación de la importancia potencial de las dunas como proveedores de beneficios. | | | | | |
| --- | --- | --- | --- | --- | --- |
| Dunas | Nula | Baja | Media | Alta | Máxima |
|  |  |  |  |  |  |

| 10. Evalúe las siguientes subcategorías de ES proporcionadas por las dunas en una escala del 1 al 5. | | | | | | |
| --- | --- | --- | --- | --- | --- | --- |
| Categorías | Subcategorías | Jerarquía | | | | |
|  |  | 1 | 2 | 3 | 4 | 5 |
| Provisioning | Hábitat para flora y fauna |  |  |  |  |  |
|  | Producción o importancia socio-económica |  |  |  |  |  |
|  | Reserva de arena |  |  |  |  |  |
|  | Espacio para almacenar minerales |  |  |  |  |  |
|  | Lugar de acumulación de material (sedimentación) |  |  |  |  |  |
| Regulation and maintenance | Regulación de procesos naturales |  |  |  |  |  |
|  | Amortigua la energía del oleaje |  |  |  |  |  |
| Cultural | Regulación hídrica (reservorio de agua) |  |  |  |  |  |
|  | Información y cultura, identidad (sentido de lugar) |  |  |  |  |  |

| 11. ¿Crees que la restauración y / o mantenimiento de las dunas es importante? |
| --- |
| a) Si ___  b) No ___  R:______________________________________________________________________  ______________________________________________________________________ |

1. **VALOR CULTURAL – POLITICO DE LAS DUNAS**

| 12. ¿Cuáles son los principales grupos humanos que se benefician de las dunas? |
| --- |
| a) Residentes locales ___  b) Pequeños agricultores ___  c) Pescadores artesanales ___  d) Comunidades mapuche ___  e) Turistas y empresas turísticas ___ |

| 13. ¿Cuáles son los principales problemas socio territoriales relacionados con las dunas? |
| --- |
| a) Conflicto de propiedad de la tierra ___  b) Extracción de materiales y áridos ___  c) Actividad turística v/s agricultura y/o ganadería ___  d) Eventos de Riesgo Naturales (tormentas, marejadas, oleajes, tsunami) ___  e) No reconoce ___ |

| 14. ¿Existen prácticas culturales que tengan lugar en las dunas y alrededores? ¿Puede mencionar alguna? |
| --- |
| a) Si ___  b) No ___  R: _____________________________________________________________________ |

| 15. ¿Ha oído hablar de la iniciativa de CONAF para crear "barreras de vegetación natural", llevada a cabo en las dunas alrededor de Saavedra después del tsunami de 2010? |
| --- |
| a) Si ___  b) No ___ |

| 16. ¿Cree que la iniciativa de CONAF (Pregunta 15) ayudaría a mitigar posibles eventos naturales asociados a tormentas, marejadas o fuertes tempestades? |
| --- |
| a) Si ___  b) No ___ |

1. **BIOFÍSICO**

| 17. ¿Sabe si hay animales que vivan en las dunas? ¿Puede usted nombrarlos? |
| --- |
| R: ______________________________________________________________________  ______________________________________________________________________ |

| 18. ¿Sabes si hay plantas que crezcan en las dunas? ¿Puede usted nombrarlas? |
| --- |
| R: ______________________________________________________________________  ______________________________________________________________________ |

| 19. Si un evento natural asociado con tormentas, marejada o fuerte oleaje afectara la costa de la Región de la Araucanía, ¿Ayudarían las dunas a mitigar los posibles impactos? |
| --- |
| a) Si ___  b) No ___ |

| 20. ¿Cree que las dunas ayudarían a mitigar posibles eventos naturales asociados con un tsunami? |
| --- |
| a) Si ___  b) No ___ |
